# Structure, Composition and Metagenomic Profile of Soil Microbiomes Associated to Agricultural Land Use and Tillage Systems in Argentine Pampas

**DOI:** 10.1371/journal.pone.0099949

**Published:** 2014-06-12

**Authors:** Belén Carbonetto, Nicolás Rascovan, Roberto Álvarez, Alejandro Mentaberry, Martin P. Vázquez

**Affiliations:** 1 Instituto de Agrobiotecnología de Rosario (INDEAR), Predio CCT Rosario, Santa Fe, Argentina; 2 Facultad de Agronomía, Universidad de Buenos Aires, Buenos Aires, Argentina; 3 Departamento de Fisiología y Biología Molecular y Celular, Facultad de Ciencias Exactas y Naturales, Universidad de Buenos Aires, Buenos Aires, Argentina; UC Irvine, United States of America

## Abstract

Agriculture is facing a major challenge nowadays: to increase crop production for food and energy while preserving ecosystem functioning and soil quality. Argentine Pampas is one of the main world producers of crops and one of the main adopters of conservation agriculture. Changes in soil chemical and physical properties of Pampas soils due to different tillage systems have been deeply studied. Still, not much evidence has been reported on the effects of agricultural practices on Pampas soil microbiomes. The aim of our study was to investigate the effects of agricultural land use on community structure, composition and metabolic profiles on soil microbiomes of Argentine Pampas. We also compared the effects associated to conventional practices with the effects of no-tillage systems. Our results confirmed the impact on microbiome structure and composition due to agricultural practices. The phyla *Verrucomicrobia, Plactomycetes, Actinobacteria*, and *Chloroflexi* were more abundant in non cultivated soils while *Gemmatimonadetes, Nitrospirae* and WS3 were more abundant in cultivated soils. Effects on metabolic metagenomic profiles were also observed. The relative abundance of genes assigned to transcription, protein modification, nucleotide transport and metabolism, wall and membrane biogenesis and intracellular trafficking and secretion were higher in cultivated fertilized soils than in non cultivated soils. We also observed significant differences in microbiome structure and taxonomic composition between soils under conventional and no- tillage systems. Overall, our results suggest that agronomical land use and the type of tillage system have induced microbiomes to shift their life-history strategies. Microbiomes of cultivated fertilized soils (i.e. higher nutrient amendment) presented tendencies to copiotrophy while microbiomes of non cultivated homogenous soils appeared to have a more oligotrophic life-style. Additionally, we propose that conventional tillage systems may promote copiotrophy more than no-tillage systems by decreasing soil organic matter stability and therefore increasing nutrient availability.

## Introduction

Agriculture is facing major challenges nowadays. Production will have to double in the next 50 years in order to face growing food demand and bioenergy needs [1], [2]. This must be done without increasing environmental threats such as climate change, biodiversity loss and degradation of land and freshwater. Achieving such a goal represents one of the greatest scientific challenges ever. This is in part because of the trade-offs among economic and environmental goals and because of the insufficient knowledge about the biological, biogeochemical and ecological processes that are relevant for sustainable ecosystem functioning [3], [4]. Much has been done during the last decade to gain sufficient information on agricultural ecosystem biology, still, more work needs to be done to gain deeper comprehension and to be able to reduce the negative environmental impacts of agriculture [2], [5], [6]. The main focus should be oriented to soil degradation. Soil fertility, as the capacity to sustain abundant crop production, needs to be preserved. Nowadays soil fertility is maintained by dependence on external inputs; with increasing water contamination [7]. In this context, the key to understand the behavior of life-supporting elements in soil, such as carbon, nitrogen, and phosphorus lies in the fluxes between their various forms in the environment, which are modulated by biology [8]. Comprehension of soil microorganism dynamics is then essential to understand soil processes that affect fertility. Ecological approaches are being taken into account in soil microbial studies trying to address these questions. These approaches involve diversity and functional analyses of soil communities [9], [10]. Scholes & Scholes point out that this complex view is necessary for the comprehension of soil systems and that soil restoration of biological processes is the key to achieving lasting food and environmental security [8].

Argentine Pampas is an important player in this scenario. With a plain area of 50 million ha., nearly 50% of the whole Pampas area is devoted to crop production [11]. Cultivation began in the 19th Century in the central humid portion of the region, in soils of high fertility, and spread in last decades to the south and the semiarid west [12]. Soil degradation (i.e. intense erosion and net loss of nutrients and organic carbon) caused by the use of conventional tillage systems were reported in the Pampas [13]–[17]. Nowadays between 60 and 80% of production is conducted under conservational no-till practices [18]. Extensive research was done to evaluate the effects of reduced tillage and no-tillage systems on soil physical properties, water content, fertility and crop yields[19]. The main outcome of these analyses points that the adoption of limited tillage systems led to soil improvement, by augmenting organic matter content and soil structure. Still, external fertilization is needed in order to restore nutrient levels and fertility regardless the tillage system employed.

Even though the effects of different tillage practices on soil physical and chemical characteristics have been deeply studied, changes in microbial biodiversity and functioning have been poorly reported in Argentinean Pampas. Most works have studied tillage effects on microbial biomass or specific microbial activities (i.e. utilization of specific substrates, extracellular enzyme production, mineralization, etc.) rather than on full microbiome [13], [20], [21]. Other studies have focused on the behavior of specific bacterial taxa [22], [23]. Reports with an ecological approach (i.e. microbial community analysis) have usually focused on individual effects of land use such as the application of herbicides [24], [25]. In these cases, biodiversity variability has been assessed using classical fingerprinting techniques (such as RFLP and DGGE) that lack information about microbial taxonomic identity and only capture the most dominant species in the environment [26], [27]. In the last few years, 16S amplicon pyrosequencing has been largely implemented to determine microbial diversity and structure of many different ecosystems worldwide [28], [29]. This strategy allows a more exhaustive characterization of community patterns and composition. Moreover, some works have incorporated the use of shotgun metagenomics to study the metabolic potential of soil microbiomes [10], [30]. The shotgun approach generates a massive amount of data using random high-trhoughput sequencing of soil isolated DNA. This allows the identification of functional capabilities by gene annotation and the comparison of metabolic profiles between samples. To our knowledge, Figuerola et al. [31] were the only authors studying microbial communities in agronomical soils of Argentine Pampas using high throughput sequencing approaches. They observed differences in microbial community composition of soils under no-tillage systems using 16S pyrosequencing. As a novelty, our efforts focused on assessing the impact of long-term agriculture on Pampas soil microbiomes using both shotgun metagenomics and deep 16S amplicon sequencing approaches. We evaluated the effect of more than a hundred years of agronomical land use on both community features and metabolic profiles of soil microbiomes in comparison with nearby control soils with no agricultural records. We also addressed the differences between the effects of two tillage systems: conventional tillage vs. no-tillage on microbial communities.

Several previous studies of soil microbiomes from different parts of the world showed the effects of agronomical land use on soil microbial communities [10], [32]–[35]. Some of these studies showed differences in trophic strategies between microbial communities related to tillage; and most of them were done in experimental plots. As a novelty, we tested the impact of long term agriculture in soils sampled in production fields in the Argentine Pampas, allowing a deeper insight to the effects of intense land use on soil ecosystems functioning. We confirmed the hypothesis that agronomical practices affected Pampas soil microbiomes by promoting a shift of life-history and trophic strategies. We also showed differences in the effects of contrasting tillage systems (i.e. conventional vs. no- tillage) on community taxonomic and metabolic composition on a long term experiment.

## Materials and Methods

### Sites description and sampling

Soil samples were taken in production and experimental fields between June and August 2010. To address the effects of agricultural land use on soil microbial communities, three different production farms were sampled in the Rolling Pampas area: “La Estrella”, “La Negrita” and “Criadero Klein” (See Rascovan et al and Table S1 for details). Rolling Pampas soils are classified as Typic Argiudolls [36] and mean annual rainfall and temperature were1002 mm and 16.8°C respectively. Two treatments were defined: *cultivated* for production plots, and *no cultivated* for farm-housés parks. Production plots were under cultivation for at least one century under conventional tillage systems, with a mixed rotation of pastures and annual grain crops. During the last 15 years before sampling plots were subjected to continuous crop cultivation under no-tillage systems (i.e. minimal soil disturbance, permanent soil cover, rotations and fertilization). The last crop rotation before sampling was wheat-soybean. Nitrogen and phosphorus fertilizers were applied. Samples were collected one month after soybean harvest. Soil samples were also collected nearby the farmers' houses where no agricultural land use (no tillage nor cultivation) was recorded for the last 30 years except from grass mowing. Parks around farmers' houses are usually considered as undisturbed environments in Argentine Pampas [37]. Soils under no land use were covered with grass and other herbaceous (non-woody) plants common in the region such as *Cirsium sp, Trifolium sp, Micropsis sp, Festuca sp, Dichondra sp, Cyperus sp* and *Taraxacum officinale*. For numerical analyses purposes the three farms are treated as experimental replicates. Four soil samples were taken with an auger from the upper 20 cm soil layer in each farm and treatment. A total of 24 samples were collected in Rolling Pampa soils.

In order to compare effects of contrasting tillage systems, samples were also collected in a 34-year-old experiment located in Balcarce in the Southern Pampas (See Rascovan et al and Table S1 for more details). Samples were taken in experimental plots because no production fields are using conventional tillage for crop production nowadays in the Pampas. Soils in Balcarce are a complex of Typic Argiudolls and Petrocalcic Paleudols and mean annual rainfall and temperatures were 875 mm and 13.8°C respectively. The experiment was carried out in three (175 m^2^) experimental plots (n = 3). Treatments were defined as: *no tillage* (NT) and *conventional tillage* (CT). NT plots had minimal soil disturbance and permanent soil cover combined with rotations; which have included pastures and grain crops (soybean, corn, wheat) during the last 16 years. CT plots were managed with moldboard plough. Nitrogen fertilization was performed in NT and CT plots (60 kg N ha-1). Last rotation before sampling was corn-soybean. Two sub-samples were collected from all treatment and replicate plots a month after soybean harvest. A total of 12 samples were collected.

Samples were immediately sent to the lab after collection. Samples used for DNA purification were air dried and sieved through 1 mm mesh to thoroughly homogenize, break aggregates and remove roots and plant detritus, then stored at −80°C. DNA purification and library preparation was previously described in Rascovan. et al.[38].

None of the sampling sites is located in protected areas. Permissions were obtained directly from each farm owner or manager: Alejandro Cattaneo at La Negrita and La Estrella, Roberto Klein at Criadero Klein and Guillermo Studdert at Balcarce experiment.

### Soil chemical and physical measurements

Soil organic carbon was determined by wet digestion and organic matter was estimated [39]. Nitrate-nitrogen was analyzed by 2 M KCL extraction and the phenoldisulfonic acid method [40]. Extractable phosphorus was determined by the Bray method [41]. The pH was measured in a soil:water ratio 1:2.5. Salinity was estimated by the determination of electrical conductivity [42]. Texture analysis was performed by the hydrometer method [43] and nitrogen was determined by Kjeldahl method [40].

### 16S amplicon sequencing and shotgun metagenomic datasets

To analyze the effect of agronomical practices on soil microbiomes, sequence data from the previously reported Pampa dataset [38] was used. In order to evaluate microbial community structure and taxonomic composition, a total of 112,800 high-quality filtered 16S rRNA gene amplicon sequences, obtained from the 42 soil DNA samples (replicates and subsamples were included). DNA shotgun metagenomic data was used to analyze metabolic profiles. Shotgun metagenomic data completed a total of 10,445,170 sequences. In this case sequences were obtained from one subsample per sampling replicated plot.

In brief, libraries were prepared as follows: DNA was isolated from 10 g of soil of each of the 42 soil samples using the Power MaxSoil DNA Isolation Kit following the manufacturer's instructions (MO BIO Laboratories, Inc.). For amplicon libraries the V4 hyper variable region of the 16s rRNA gene was amplified. Duplicated reactions were performed using barcoded bacterial universal primers containing Roche- 454 sequencing A and B adaptors and a nucleotide multiple identifier (MID) to sort samples: 563F: 5′-CGTATCGCCTCCCTCGCGCCATCAGACGAGTGCGTAYTGGGYDTAAAGNG -3′ (where ACGAGTGCGT is an example, different MIDs for each sample were used) and 802R (5′-CTATGCGCCTTGCCAGCCCGCTCAGTACCRGGGTHTCTAATCC, 5′-CTATGCGCCTTGCCAGCCCGCTCAGTACCAGAGTATCTAATTC, 5′-CTATGCGCCTTGCCAGCCCGCTCAGCTACDSRGGTMTCTAATC, 5’-CTATGCGCCTTGCCAGCCCGCTCAGTACNVGGGTATCTAATCC) [44]. All amplicons were cleaned using Ampure DNA capture beads (Agencourt- Beckman Coulter, Inc.) and pooled in equimolar concentrations before sequencing on a Genome Sequencer FLX (454-Roche Applied Sciences) using Titanium Chemistry according to the manufacturer's instructions.

Shotgun metagenomic libraries were prepared by nebulization, followed by tagging with GS-FLX-Titanium Rapid Library MID Adapters Kit (454-Roche Applied Sciences) and sequenced with a Genome Sequencer FLX (454-Roche Applied Sciences) using Titanium Chemistry according to the manufacturer's instructions. Sequencing runs were performed in INDEAR sequencing facility.

All the sequences used in the present study are available in The Sequence Read Archive (SRA) under accession number SRA058523 and SRA056866. See Rascovan et al. [38] for more information.

### Amplicon sequence processing, OTU classification and taxonomic assignment

Sequence data were quality controlled and denoised with the ampliconnoise.py script of QIIME [45].This script also eliminated chimeras. Sequences obtained from Rolling Pampa soil libraries and Balcarce soil libraries were processed separately. Sequences were clustered into Operational Taxonomic Units (OTUs) using the pick_otus.py script with the Uclust method [46] at 97% sequence similarity. Rolling Pampas samples yielded 2,591sequences on average (ranging from 1,455 to 3,991sequences). Balcarce samples yielded 2,329 reads on average (ranging from 1,211 to 4,755 reads).OTU representative sequences were aligned using PyNast algorithm [47] with QIIME default parameters. Phylogenetic trees containing the aligned sequences were then produced using FastTree [48]. All downstream analyses were determined after each sample was randomly rarefied to 70% the number of reads of the smallest sample (i.e. 1,080 reads for Rolling Pampa libraries and 850 reads for Balcarce libraries). Phylogenetic distances between OTUs were calculated using unweighted and weighted Unifrac [49]. Taxonomic classification of sequences was done with Ribosomal Database Project (RDP) Classifier using Greengenes database using a 50% confidence threshold [50], [51].

### Microbial community analyses

Unifrac phylogenetic pairwise distances among samples were visualized with principal coordinates analysis (PCoA). Analysis of similarity statistics (ANOSIM) was calculated to test a-priori sampling groups. BIOENV analysis was performed to elucidate which soil properties correlated with community patterns. All calculations were carried out with R packages ‘BiodiversityR’ and ‘Vegan’ [52], [53]. T- tests were performed with QIIME script otu_category_significance.py, and R scripts in order to elucidate differences in read abundances.

### Shotgun metagenomic sequence processing and analysis

SSF files obtained from shotgun sequencing runs were uploaded to the MG-RAST webserver [54] for sequence filter and analyses. Reads more than two standard deviations away from the mean read length were discarded. For dereplication removal MG-RAST used a simple k-mer approach to rapidly identify all 20 character prefix identical sequences. This step is required in order to remove artificial duplicate reads. We obtained an average of 1.28×10^6^ filtered reads per sample for Rolling Pampa shotgun libraries, and an average of 304,258 filtered reads per sample for Balcarce libraries.

Filtered high quality sequences were assigned to Cluster of orthologous groups (COG) by the MG-RAST sever pipeline using a similarity-based approach. COGs were assigned with a maximum E value of 10^−20^, an average alignment of 80 amino acids length and 70% average identity.

Relative abundances were calculated by dividing the number of hits for each COG or COG-category by the total number of filtered reads in each sample. Euclidean distances based on relative abundances were calculated between sample pairs. PCoA visualizations and ANOSIM calculations were performed. All calculations were carried out with R packages ‘BiodiversityR’ and ‘Vegan’. T- tests were performed with QIIME script otu_category_significance.py, and R scripts in order to elucidate differences in COG relative abundances between samples.

## Results

### Microbiome community changes related to agricultural land use

**Figure 1:**
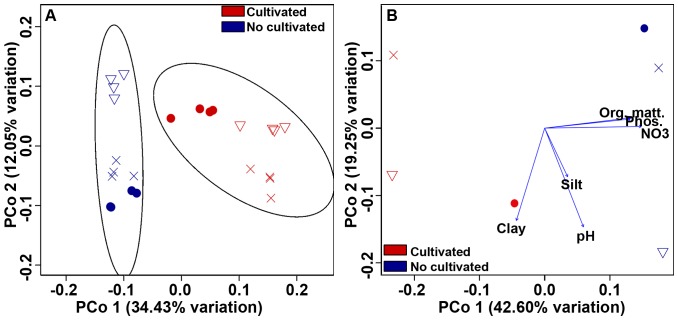
PCoA plots of Pampa production field soil microbiomes based on 97% similarity Weighted Unifrac distance matrices. A) PCoA of cultivated and non-cultivated soil microbiomes. All sub-samples are plotted. Standard error ellipses show 95% confidence areas. B) PCoA biplot of soil properties that best explained variation in community structure. Correlations were calculated using BIOENV on average data of each sampled site (Mantel r = 0.6107, p≤0.05). Circles represent samples from “La Estrella”, crosses represent “Criadero Klein” samples and triangles represent “La Negrita” samples.

The PCoA visualization revealed clear differences between cultivated and noncultivated soils (ANOSIM R = 0.8406, p≤0.001; Figure 1A).Similar results were obtained when using Bray Curtis distance matrices (Figure S1).

The soil properties (Table S1) that best explained the phylogenetic variation observed in microbial communities were determined using Clarke and Ainsworth's BIOENV analysis. Our results showed that variables that best correlated with community differences were organic matter, clay and silt content, nitrates, phosphorus and pH (Mantel r = 0.6107, p≤0.05).The PCoA biplot (Figure 1B) showed that organic matter, phosphorus and nitrate levels correlated with the first ordination axis that discriminates between cultivated and non cultivated soils. These three properties were higher in soils under no land use.

**Figure 2:**
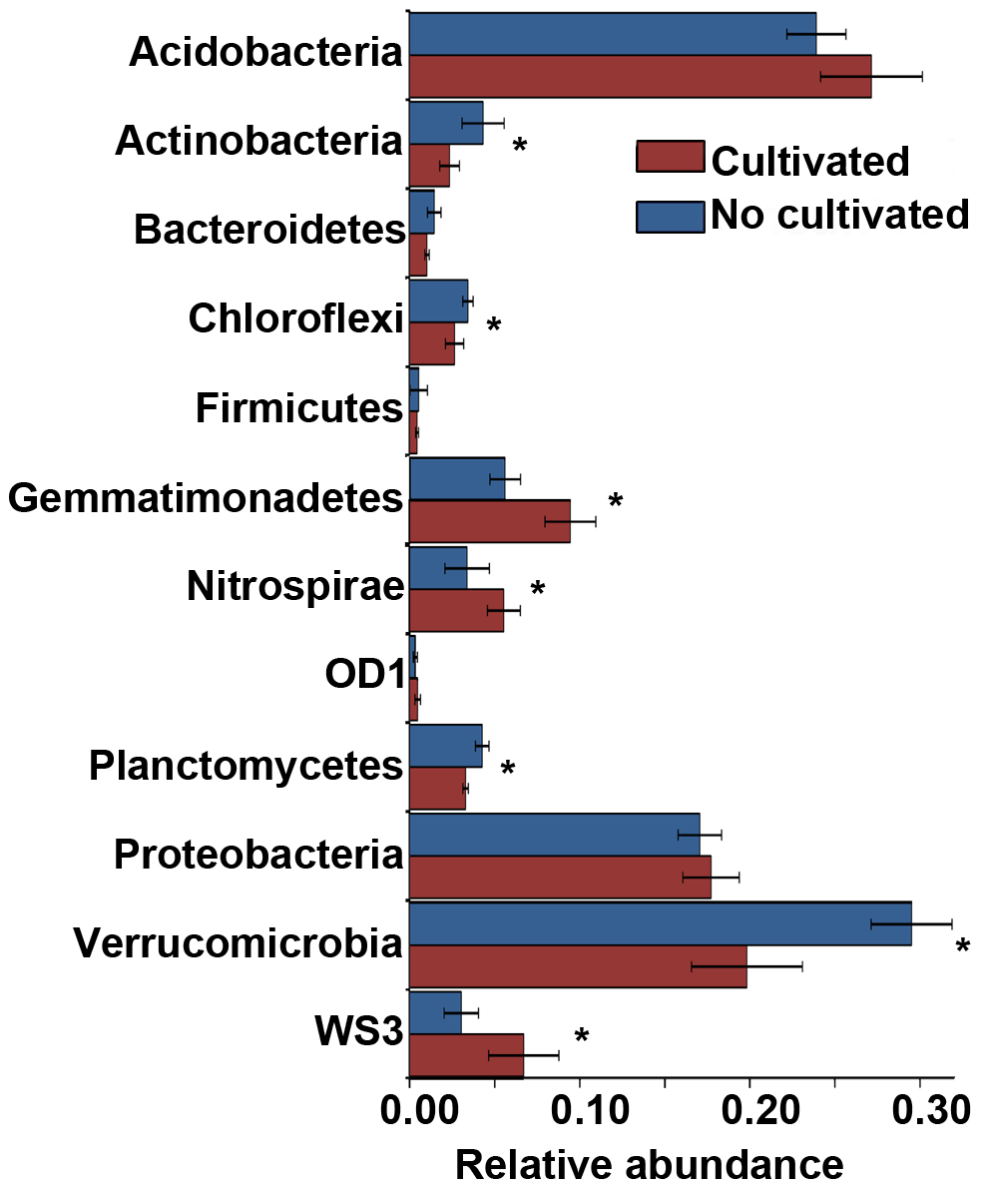
Relative abundances of taxonomic groups in Pampa production field soil microbiomes. Bars represent ± 1 standard error. (*) indicate significant differences (p≤0.05).

Regarding the taxonomic analyses, we observed that members of phyla *Verrucomicrobia, Planctomycetes, Actinobacteria* and *Chloroflexi* were more abundant in non cultivated soils (p≤0.05) (Figure 2). On the other hand, we found that sequences related to *Gemmatimonadetes*, candidate division WS3 and *Nitrospirae* were enriched in cultivated soils (p≤0.05) (Figure 2). No significant differences were found for *Proteobacteria* (for non of the Clases), *Acidobacteria* and *Bacteroidetes* phyla. The ten mentioned taxa represent on average 95% of total sequences of each sample.

### Microbiome metagenomic profile changes related to agricultural land use

**Figure 3:**
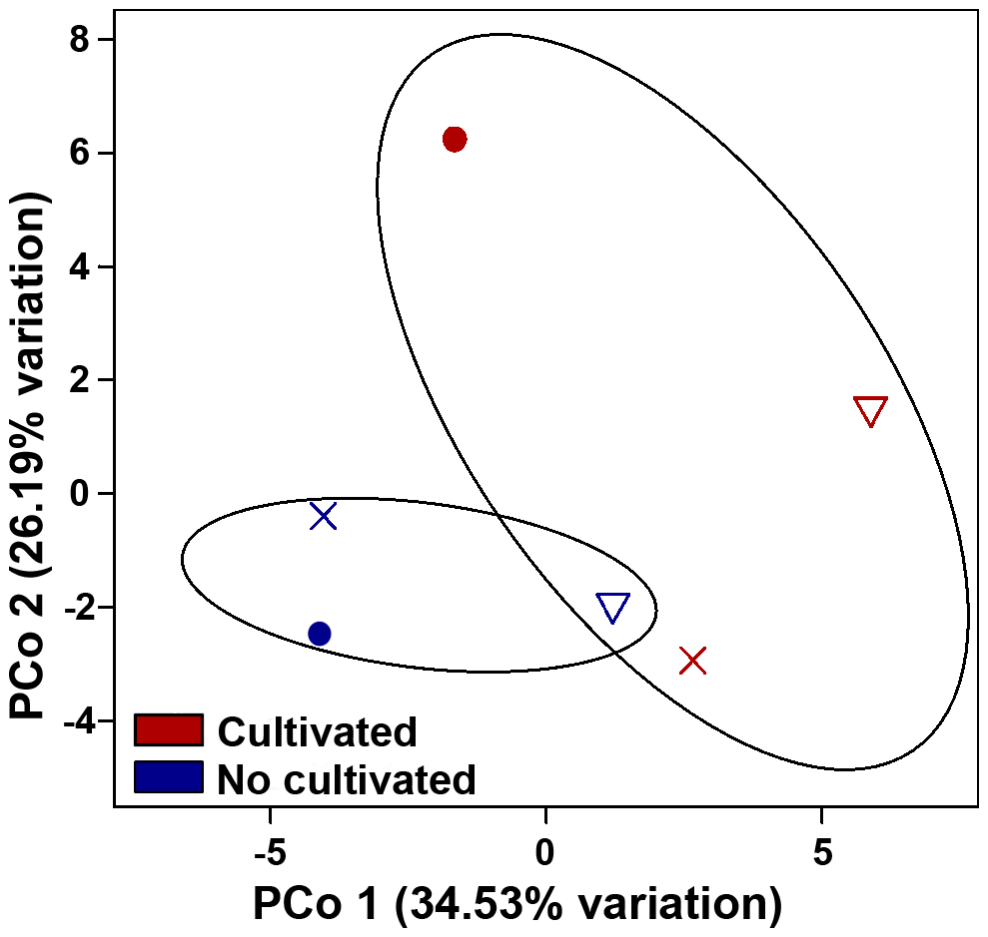
PCoA plot of metagenomic data based on Euclidean distance matrices of COG categories of Pampa production field soil microbiomes. Standard error ellipses show 95% confidence areas. Circles represent samples from “La Estrella”, crosses represent “Criadero Klein” samples and triangles represent “La Negrita” samples.

**Figure 4:**
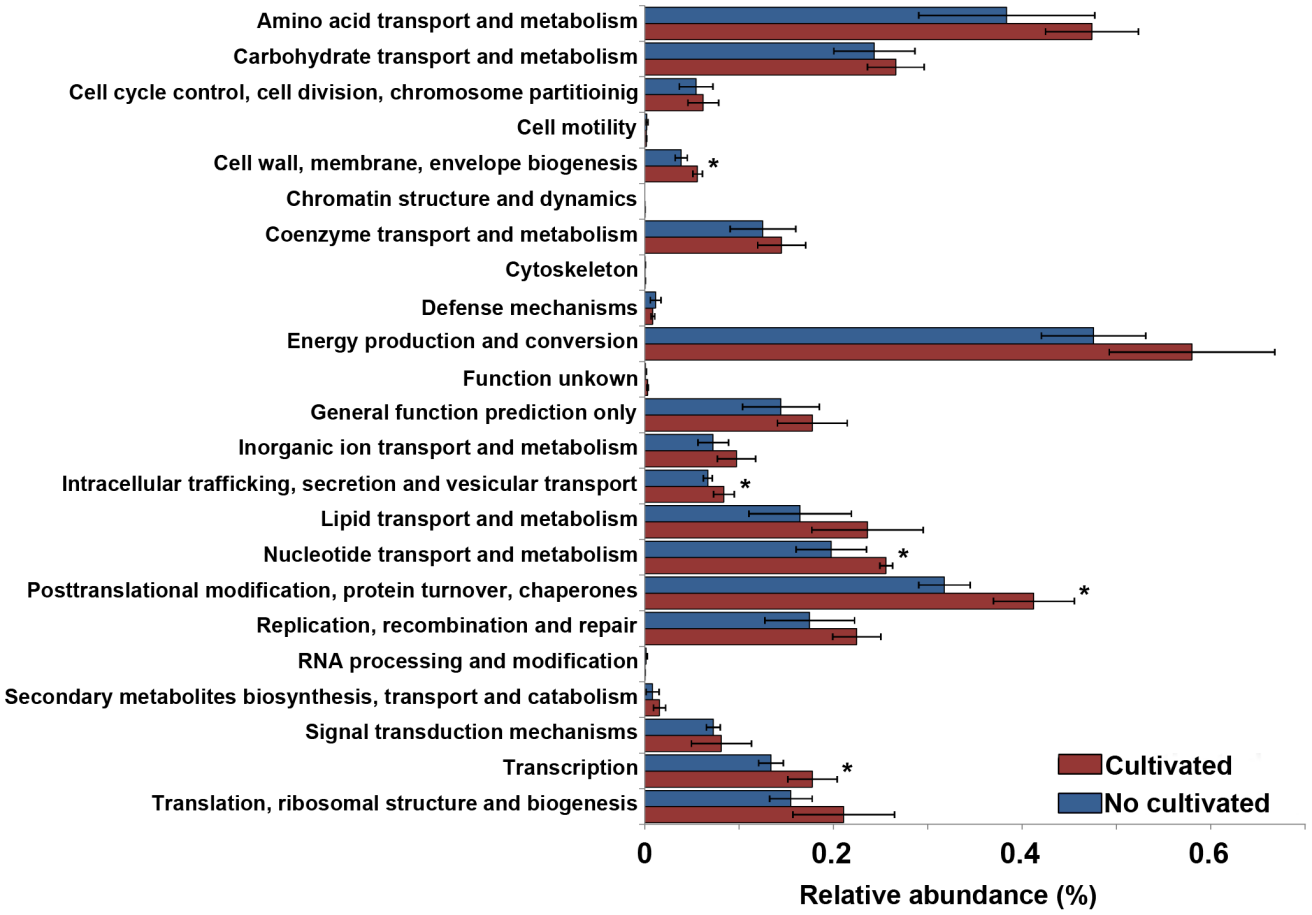
Relative abundances of COG categories in Pampas production field soil microbiomes. Bars represent ±1 standard error. (*) indicate significant differences (p≤0.05).

We found that cultivated and non cultivated soils also clustered apart when metagenomic functional categories were used for the analysis. The first two components of the PCoA explained over 60% of the variability between samples (Figure 3). Standard deviation ellipses overlapped in the ordination plot, indicating that some features are shared between metagenomes. Still, a positive correlation was observed between metabolic and weighted-Unifrac distance matrices (Mantel r: 0.5036 p≤0.05). The analyses of individual COG categories revealed that the relative abundances of COG categories associated with transcription, protein modification, nucleotide transport and metabolism, wall and membrane biogenesis and intracellular trafficking and secretion were higher in cultivated soils (Figure 4, p≤0.05). A deeper analysis inside COG categories revealed that COGs related to Coenzyme A and acetyl-Coa metabolism, energy storage and starvation or quiescence such as, pantothenate kinase, phospho-transacetylase, and trehalose utilization protein were more abundant in non cultivated than in cultivated soils (Figure S2, p≤0.05). On the other hand, COGs related to rapid regulation systems, tricarboxylic acid cycle and nitrogen assimilation such as urease, citrate synthase, glutamate synthase, fumarate hydratase, S-adenosyl- homocysteine hydrolase, S-adenosyl-methionine synthetase, cobalamin biosynthesis protein and ABC transporters, were more abundant in cultivated soils (Figure S2, p≤0.05).

### Microbiome community structure and composition related to conventional tillage and no-tillage systems

**Figure 5:**
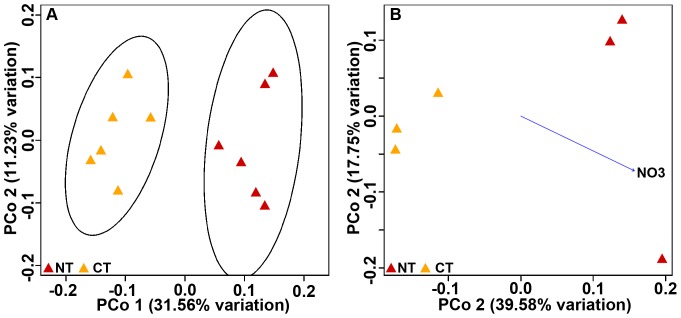
PCoA plots of Balcarce experimental field soil microbiomes based on 97% similarity Weighted Unifrac distance matrices. A) CA: conventional tillage; NT: no-tillage. All sub-samples are plotted. Standard error ellipses show 95% confidence areas. B) PCoA biplot, nitrate was the variable that best explained variation in community structure. Correlations were calculated using BIONEV on average data of each experimental plot (Mantel r = 0.7721, p≤0.01).

To compare the structure of microbiomes under different tillage systems, we collected samples from an experimental field located in Balcarce in the Southern Pampas. The 34-year-old experiment compared two tillage systems: no-tillage (NT) and conventional tillage (CT). Weighted Unifrac analysis showed differences in community structure associated to the tillage system employed (ANOSIM R = 0.9009, p<0.05, Figure 5A). The first axis explained 31.56% of total variation and separated NT from CT. Additionally, we showed that nitrates were the only soil variable (Table S1) that significantly correlated with community structure (BIOENV analysis, Mantel r = 0.7721, p≤0.01, Figure 5B).

**Figure 6:**
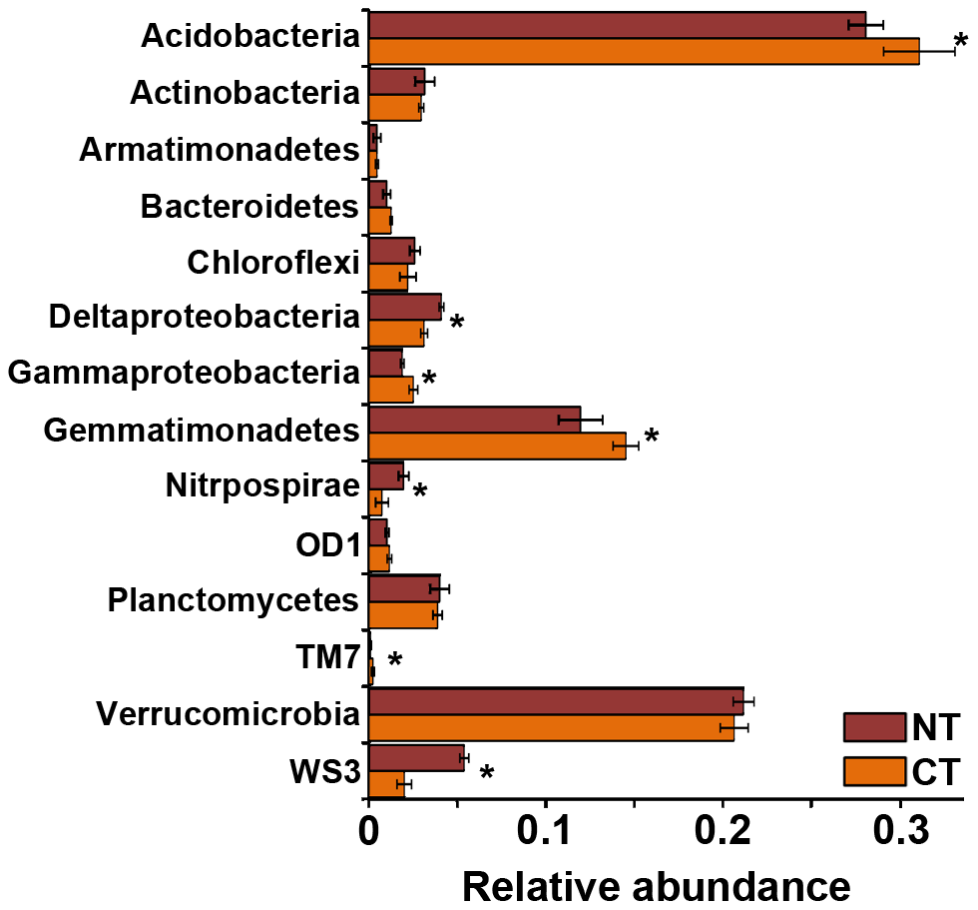
Comparison of relative abundances of taxonomic groups in NT and CT soils. CA: conventional tillage; NT: no-tillage. Bars represent ±1 standard error. (*) indicate significant differences (p≤0.05).

Moreover, microbiomes of CT and NT soils also differed in taxonomic composition. Members of *Acidobacteria*, *Gemmatimonadetes*, candidate division TM7 and class *Gammaproteobacteria* were more abundant in CT soils, while *Nitrospirae*, candidate divisionWS3 and *Deltaproteobacteria* were more represented in NT soils (Figure 6, p≤0.05).

### Microbiome metabolic profiles related to conventional tillage and no- tillage systems

**Figure 7:**
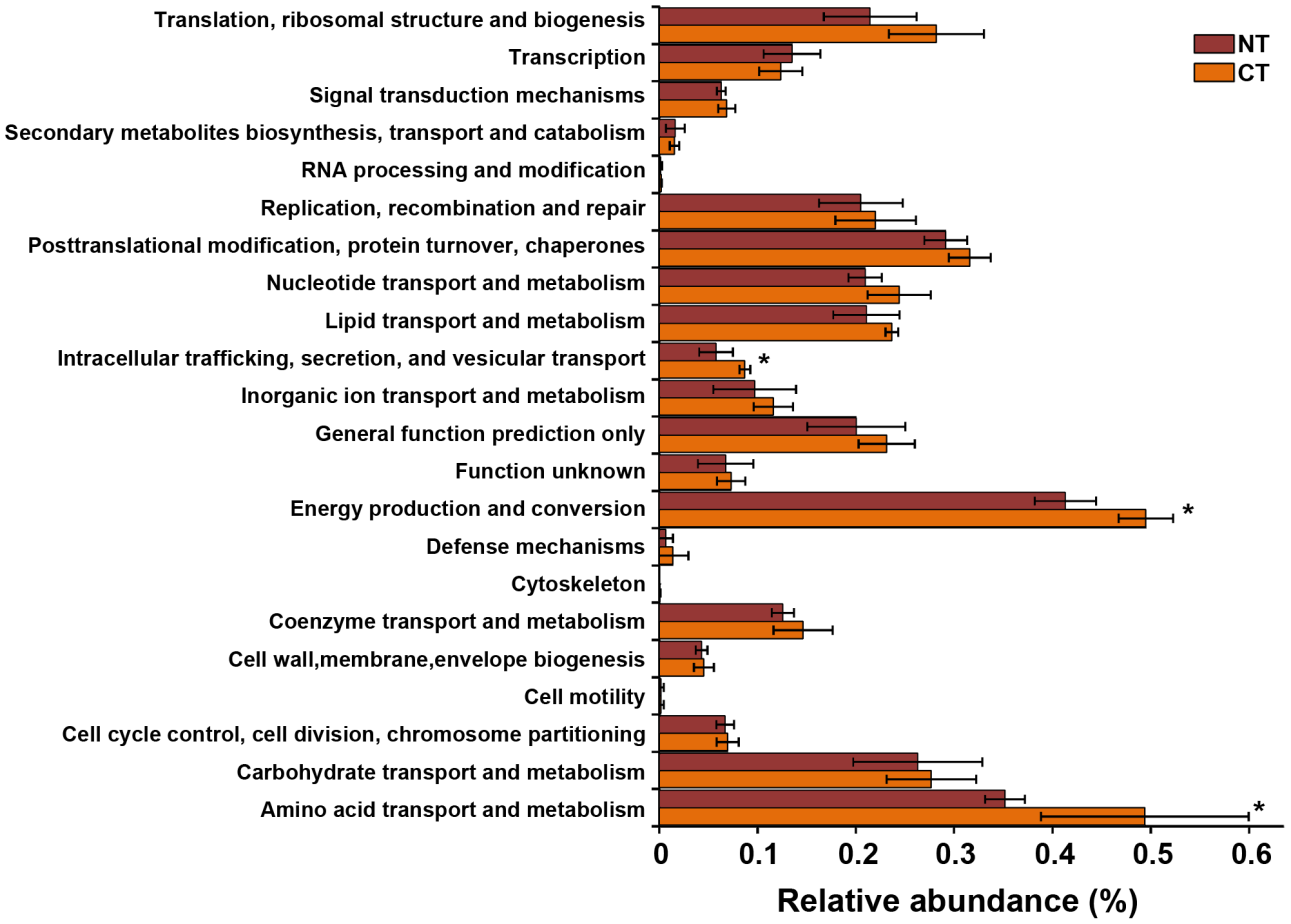
Relative abundances of COG categories in soil microbiomes of tillage systems comparison experiment in Balcarce. CA: conventional tillage; NT: no-tillage. Bars represent ±1 standard erro. (*)indicate significant differences (p≤0.05).

Variation in metagenomic profiles between CT and NT microbiomes was analyzed with PCoA based on Euclidean distance matrices of COG abundances. We could not find significant differences in overall profile metabolic structure between tillage systems (Figure S3). Additionally, we did not find significant correlation between Euclidean metabolic matrices and phylogenetic matrices. However, categories related to intracellular trafficking and secretion, amino acid transport and metabolism, and energy production and conversion were shown to be more abundant in soil under CT (Figure 7).

## Discussion

Much work still needs to be done to get a comprehensive view of the soil microbiomes. Our work is one of the firsts done in the Argentine Pampas at this resolution level, with a combination of metagenomic and phylogentic approaches; and it is aimed to contribute to the comprehension of soil microbiomes function and dynamics. In that context, our results are in agreement with previous works that showed differences in soil microbial community structure and taxonomic composition due to the presence of agricultural land use [9], [10], [32], [33].

### Effects of agricultural land use on community composition of soil microbiomes in Argentine Pampas

We observed effects on taxonomic composition at the phylum level. *Verrucomicrobia, Plactomycetes, Actinobacteria* and *Chloroflexi* were more abundant in soils that were never cultivated; while *Gemmatimonadetes, Nitrospirae* and WS3 were more abundant in crop cultivated soils. These results are in agreement with the copiotroph/oligotroph hypothesis [55], that propose that high number of oligotrophic prokaryotes may be found in bogs and soils with high amounts of recalcitrant organic matter [56]. On the other hand, copiotrophic organisms are able to use labile nutrient fractions and to grow at higher rates as a consequnece. In our study, non-cultivated soils are considered to be oligotrophic since they present high levels of organic matter highly rich in humic acids [12] and cultivated soils as copiotrophic environments due to fertilization and the seasonal presence of crop residues, which increases organic matter and nitrogen accessibility [57]. Under this assumption, bacteria in non-cultivated soils are expected to be K selected and to present low growth rates and very efficient nutrient uptake systems with higher substrate affinities. In contrast, bacteria in cultivated soils are expected to be r-selected and to have higher rates of activity per biomass unit, higher turnover rates and faster growth rates. Our results showed a trend toward these statements since a reduction in the abundance of taxa with oligotrophic characteristics, such as *Verrucomicrobia* [34], and *Planctomycetes* [58] were detected in cultivated fertilized soils. Moreover, this is in agreement with recent findings that confirm a correlation between *Verrucomicrobia* abundance patterns and conditions of limited nutrient availability in Prairie Soils in the United States [9]. On the other hand, the relative abundance of phylum *Gemmatimonadetes* was increased in fertilized cultivated soils as previously described for nitrogen-fertilized forest soils [59]. Consistently with our results, these authors observed that nitrogen fertilization was related to a higher abundance of *Gemmatimonadetes* and detected no presence of *Verrucomicrobia*. Little is known about *Gemmatimonadetes* ecology and metabolism since only one representative from this phylum has been isolated and characterized [60]. Even though, their presence in environments with a wide range of nutrient concentrations and redox states suggests versatile metabolisms [61].

We can also say that cultivated soils are more heterogeneous environments than non cultivated soils. Crop rotation, periodic fertilization and pesticide application generate temporal and spatial changes in soil chemical properties, and therefore in nutrient availability and approachability for microorganisms. Bacteria dominating in this type of soils should be adapted to heterogeneity. These microorganisms should be able to fine-tune carbon and nitrogen intakes according to their metabolic needs under frequent external changes. This seems to be the case for *Gemmatimonadetes* suggesting a generalist ecological strategy. The high abundance of *Nitrospirae* may also respond to heterogeneous conditions. It has been proposed that *Nitrospirae* lineages occupy different positions on an imaginary scale reaching from K- to r-strategies [62]. We hypothesize that in cultivated soils, *Nitrospirae* K-strategists would be exploiting nitrite in high N microenvironments, while r-strategist would be mining low concentration areas in a nitrogen gradient enhanced by fertilization [63]. This competition would be less fierce in non cultivated soils due to the highest homogeneity and a less marked nitrogen gradient due to the lack of fertilization and more recalcitrant organic matter forms.

### Effects of agricultural land use on metabolic profiles of soil microbiomes in Argentine Pampas

Our results from shotgun metagenomic data also indicated a tendency for the microbiomes of cultivated soils towards adaptation to nutrient heterogeneity. The highest relative abundances of sequences assigned to COGs related to transcription, protein modifications, nucleotide transport and metabolism, wall and membrane biogenesis and intracellular trafficking and secretion in cultivated fertilized soils are consistent with a copiotrophic strategy (i.e. rapid tight metabolism regulation and fast grow rates).Moreover, some of these COG categories were previously shown to be up-represented in copiotrophic marine microorganism genomes [58]. In a deeper look we detected that the relative abundance of sequences assigned to riboswitch regulated genes was higher in cultivated soils (i.e. cobalamin biosynthesis protein, S-adenosyl-methionine synthetase, S-adenosyl- homocysteine hydrolase) [64]–[66]. These ancient regulators may be playing a central role in a life-style strategy adapted to nutrient heterogeneity since they were described to be the most ‘economical’ and fast-reacting regulatory systems (no intermediate factors involved) [67]. Moreover, the abundant riboswitch- COGs in cultivated soils were related to synthesis of B vitamins. The levels of these vitamins have already been linked to differences in community composition in marine ecosystems [68]. More studies are needed in order to address this relationship in soil environments; still, our results suggest an important role of B-vitamins in fertilized heterogeneous soils.

Another interesting observation was the highest abundance of glutamate synthase (GOGAT) related COGs in cultivated soils metagenomic profiles. The combined role of GOGAT with glutamine synthetase and glutamate dehydrogenase allows the cells to sense ammonia external levels [69]. The high abundance of this regulation system detected in cultivated soil metagenomes suggests its importance in the detection of N fluxes related with fertilization. Moreover COGs related to the tricarboxylic acid cycle (TCA), such as citrate synthase and succinate dehydrogenase, were more abundant in cultivated soil microbiomes. GOGAT nitrogen assimilation pathway and TCA are related. It has been shown that the concentration of compounds of both pathways changes considerably and rapidly upon nitrogen up shift; in contrast, the concentrations of glycolytic intermediates remains homeostatic [70]. In addition, abundance of urease related COGs were also higher in cultivated soils. The soils sampled in this study have a long history of agricultural land use and have been long fertilized with both type of nitrogen (i.e. ammonia-based and urea-based fertilizers). This kind of environmental pressure finally selected a microbiome adapted to changing N sources and availability.

It is important to mention that these results do not infer that expression or activity of these metabolisms will be necessarily increased in cultivated soil microbiomes, still, the highest abundance of these COGs could be reflected in a highest diversification and specialization. The presence of a highest copy number of strategic genes have already been linked to copiotrophic or oligotrophic life-styles [58].

Microbiomes of non cultivated soils showed a higher abundance of sequences related to Coenzyme A and acetyl-Coa metabolism than microbiomes of cultivated soils. It is known that acetyl-CoA is a fundamental building block and energy source [71]. High acetyl-CoA levels would indicate a “proliferative” or “fed” state, while low acetyl-CoA levels (and high CoA levels) would be indicative of a “quiescent” or “starved” state. Pantothenate kinase (PanK), the key enzyme in CoA syntehsis, was also highest in non cultivated soil microbiomes [72]. In addition, it was stated that some oligotrophs preferentially use lipids as immediate and stored sources of carbon and energy in marine environments [58]. The observed abundance of Pank genes may ensure a correct CoA intracellular level in fasted moments, allowing proper lipid utilization. In addition, non cultivated metagenomic profiles showed higher abundance of sequences related to trehalose utilization. Trehalose is known to serve as energy source in many microorganisms [73]. Members of *Actinobacteria* genera *Mycobacterium* and family *Frankiaceae* are known to produce and/or utilize trehalose [74]–[76]. As mentioned above, our results showed higher abundance of *Actinobacteria*-related sequences in non cultivated soils. Moreover, sequences classified within family *Frankiaceae* were only present in these soils and the abundance of sequences assigned as *Mycobacterium* was higher than in cultivated soils (not shown). These observations are congruent with an oligotrophic strategy based on the use of storage components for carbon and energy sources.

The responses of the phylogenetic structure and metagenomic profile to agronomic land use were significantly correlated, suggesting some degree of correspondence between these different microbiome features. These results agree with previous observations done in soils from different biomes [9], [77]. Moreover, Fierer et al. found similar a similar correlation between metagenomic and phylogenetic data for microbial communities of agricultural soils under different nitrogen gradients in experimental plots [10].

### Correlation with soil properties

In addition, soil properties such as organic matter, phosphorus and nitrate levels explained most of the variability observed between cultivated and non cultivated soils. As mentioned, the highest amount of organic matter is found mostly in a recalcitrant form in these soils. Moreover, it was already established that nitrate accumulation exhibits a negative correlation with organic carbon availability [78]. In addition it has been proved that organic matter source and quality played an important role in regulating the magnitude of carbon metabolism and could be as important as nutrient abundance in water environments [79]. Our results are congruent with these observations since non cultivated soils, with highest levels of organic carbon, presented metagenomic profiles with tendencies to oligotrophy and the best explanation for this scenario is the low lability of the recalcitrant forms of carbon and nitrogen.

### Assessing the effects of different tillage systems on Pampas soil microbiomes

Our results also showed differences in the structure and composition of soil microbiomes between no-till and conventional tillage soils. Sequences related to phyla *Gemmatimonadetes*, candidate division TM7 and *Acidobacteria*, were highest in CT soils, while the abundances of *Nitrospirae* and candidate division WS3 were highest in NT soils. Tillage is the principal agent producing soil disturbance and subsequent soil structure modification [80], [81]. The negative effects of CT in soil stabilization and macroaggregate losses were previously registered in Pampas soils [82]. It has also been shown that NT increases macroaggregate abundance and organic matter content [83].These increments are related with a more recalcitrant organic matter, with increased humic acid contents and nutrient retention [84]–[86]. The higher abundance of *Nitrospirae* in NT soils reinforces the idea of a community adapted to a better N mining in these environments. Moreover, we observed very low abundance of *Nitrobacter* related sequences in Balcarce soils and no significant difference was observed between CT and NT soils (not shown). Changes in ammonia oxidation due to a decrease in ammonia availability by humic substances have already been proposed in microcosm experiments [87]. If this is the case of Balcarce soils, higher humic acids in NT soils would be decreasing ammonia availability for oxidation into nitrite and therefore decreasing nitrate availability, compared to CT soils. The predicted highest stability of organic matter and abundance of macroaggregates in NT soils are probably generating more marked nitrite gradients than in CT soils. The highest abundance of *Nitrospirae* in NT may be reflecting a highest lineage diversity that would be better adapted to these gradients.

In addition, communities under NT showed higher abundance of sequences related to the order *Syntrophobacterales* (*Deltaproteobacteria*, Figure S4). These are known anaerobic and syntrophic organisms [88], [89]. These characteristics may be an advantage in stable soils with higher number of highly humic macro aggregates since syntrophy is known to be important for community functioning in micro-environments with low nutrient levels [90]. On the other hand, CT soils presented higher relative abundance of *Gammaproteobacteria* related sequences. The order *Xanthomonadales* was the main responsible for these differences (Figure S4). This taxon has already been associated to CT practices [32].

Even though we could not find significant differences associated to tillage systems at the community structure level for metabolic profiles, COG categories related to intracellular trafficking and secretion, amino acid transport and metabolism and energy production and conversion were more abundant in CT mirobiomes. These results suggest a tendency of CT microbiomes to a more copiotrophic life-style strategy than NT microbiomes.

## Conclusion

Our results are consistent with the hypothesis that microbiomes exhibit different life- history and trophic strategies in Pampean soils under different land uses and tillage systems. Our data suggest that microbiomes of fertilized cultivated soils have more flexible metabolisms adapted to nutrient fluxes with tendencies to copiothropy while microorganisms in non cultivated soils are better adapted to lowest external nutrient availability and homogenous environment. The lowest nutrient accessibility in non cultivated soils may be explained by the higher amount of humic substances, recalcitrant organic matter and the lack of fertilizer amendments. Moreover, NT soils, with most stable structure and highest macroaggregate abundance, presented microbiomes better adapted to recalcitrant environments; while CT microbiomes presented a higher tendency to copiotrophy.

This work is of major contribution to understand how historical changes in soil properties due to agronomical land use have altered the diversity and function of below-ground communities. The importance of high-throughput characterization for the reconstruction of pre- agricultural microbiomes is being reinforced nowadays [9]. Following this direction, our findings will be very useful in future restoration and monitoring programs of Argentine Pampas ecosystems.

## Supporting Information

Figure S1 **PCoA plots of Pampa production field soil microbiomes based on average Bray Curtis distance matrices.** A) PCoA of cultivated and non cultivated soil microbiomes. Standard error ellipses show 95% confidence areas. B) PCoA biplot of soil properties that best explained variation in community structure. Correlations were calculated using BIOENV on average data of each sampled site (Mantel r = 0.6214, p≤0.05). Circles represent samples from “La Estrella”, crosses represent “Criadero Klein” samples and triangles represent “La Negrita” samples.
(PDF)

Figure S2 **Relative abundances of Cluster of Orthologous groups (COGs) in Pampa production field soil microbiomes.** Bars represent ± 1 standard error. Only significant COGs are showed.
(PDF)

Figure S3 **Comparison of tillage systems effects on the structure of metabolic profiles. PCoA plot based on Euclidean distance matrices.** CA: conventional tillage; NT: no-tillage. Standard error ellipses show 95% confidence areas.
(PDF)

Figure S4 **Relative abundances of reads assigned to orders within classes** ***Gammaproteobacteria*** **and** ***Deltaporteobacteria*** **in NT and CT soils.**
(PDF)

Table S1 **Soil chemical and physical properties.**
(XLSX)
