# Supplementary figures and images for: Structure, Composition and Metagenomic Profile of Soil Microbiomes Associated to Agricultural Land Use and Tillage Systems in Argentine Pampas
Source: PLoS One. 2014 Jun 12;9(6):e99949. doi: 10.1371/journal.pone.0099949 (PMC4055693; doi:10.1371/journal.pone.0099949)

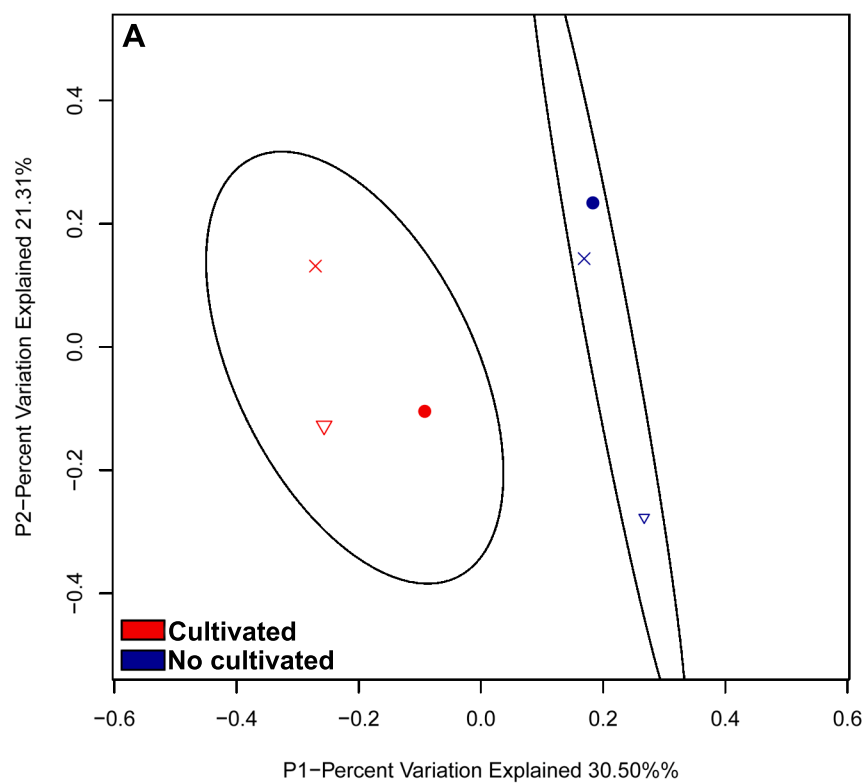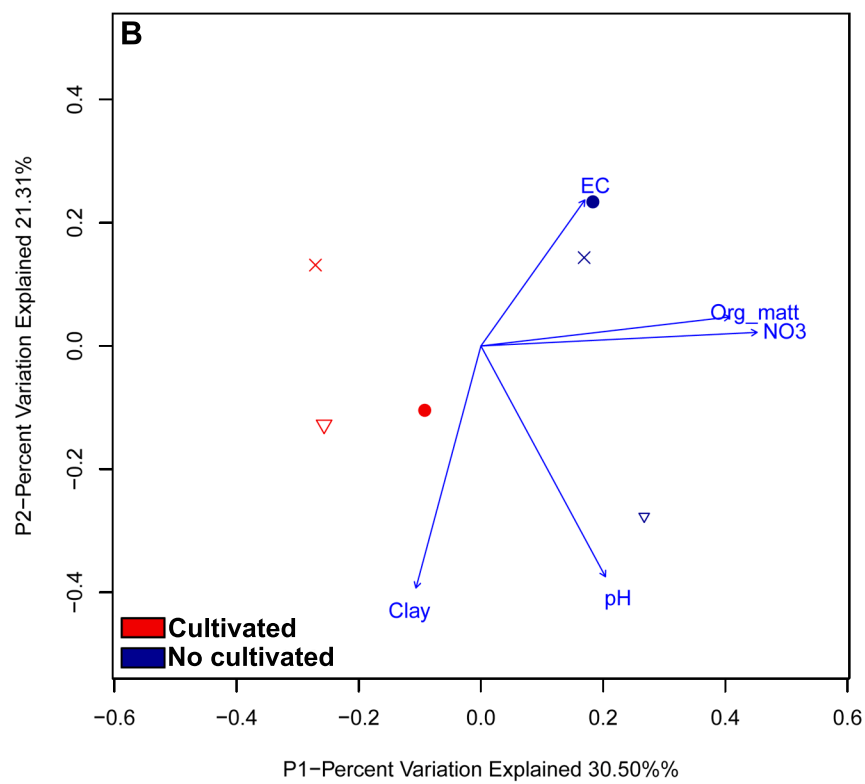

Supplement: Figure S1 — PCoA plots of Pampa production field soil microbiomes based on average Bray Curtis distance matrices. A) PCoA of cultivated and non cultivated soil microbiomes. Standard error ellipses show 95% confidence areas. B) PCoA biplot of soil properties that best explained variation in community structure. Correlations were calculated using BIOENV on average data of each sampled site (Mantel r = 0.6214, p≤0.05). Circles represent samples from “La Estrella”, crosses represent “Criadero Klein” samples and triangles represent “La Negrita” samples. (PDF) [file pone.0099949.s001.pdf]

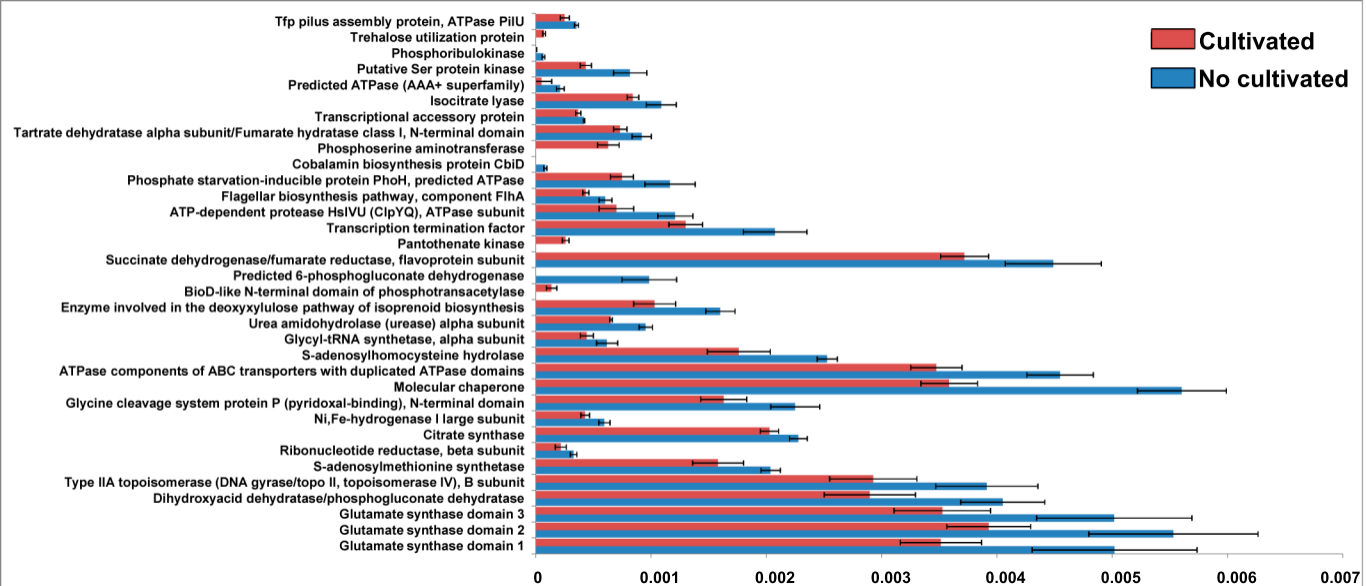

Supplement: Figure S2 — Relative abundances of Cluster of Orthologous groups (COGs) in Pampa production field soil microbiomes. Bars represent ± 1 standard error. Only significant COGs are showed. (PDF) [file pone.0099949.s002.pdf]

P2-Percent Variation Explained 26.16

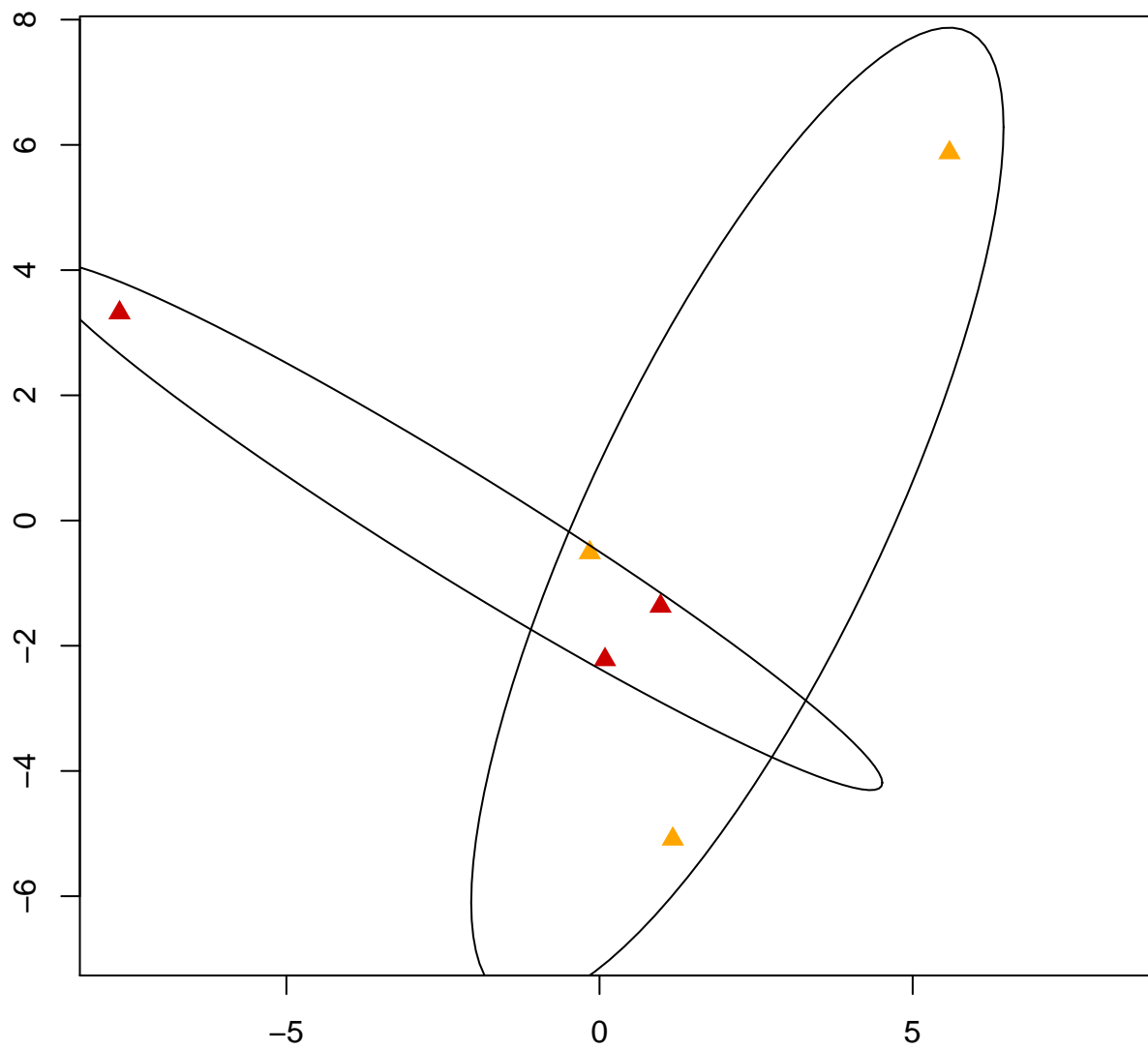

P1-Percent Variation Explained 30.77

Supplement: Figure S3 — Comparison of tillage systems effects on the structure of metabolic profiles. PCoA plot based on Euclidean distance matrices. CA: conventional tillage; NT: no-tillage. Standard error ellipses show 95% confidence areas. (PDF) [file pone.0099949.s003.pdf]

### Gammaproteobacteria

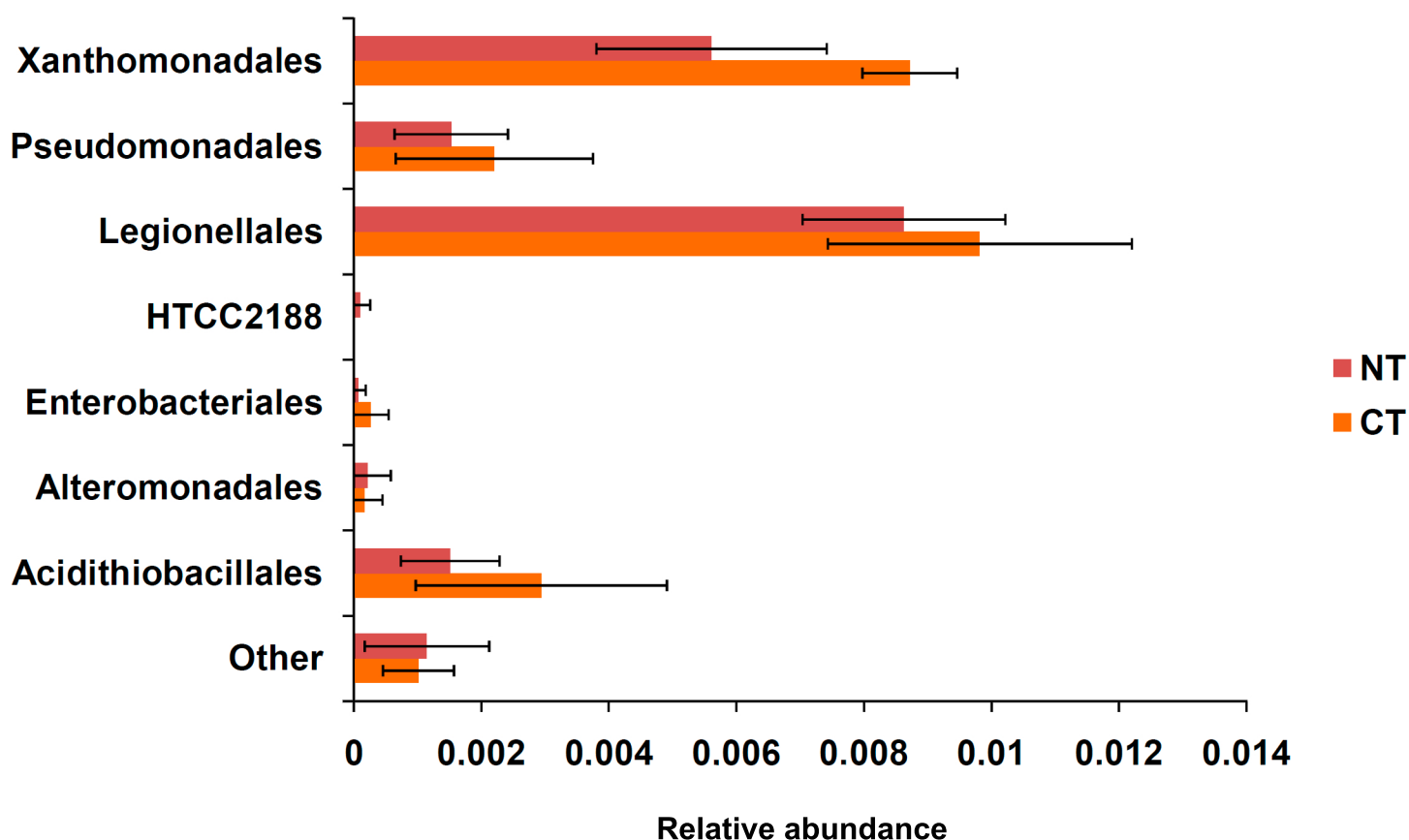

### Deltaproteobacteria

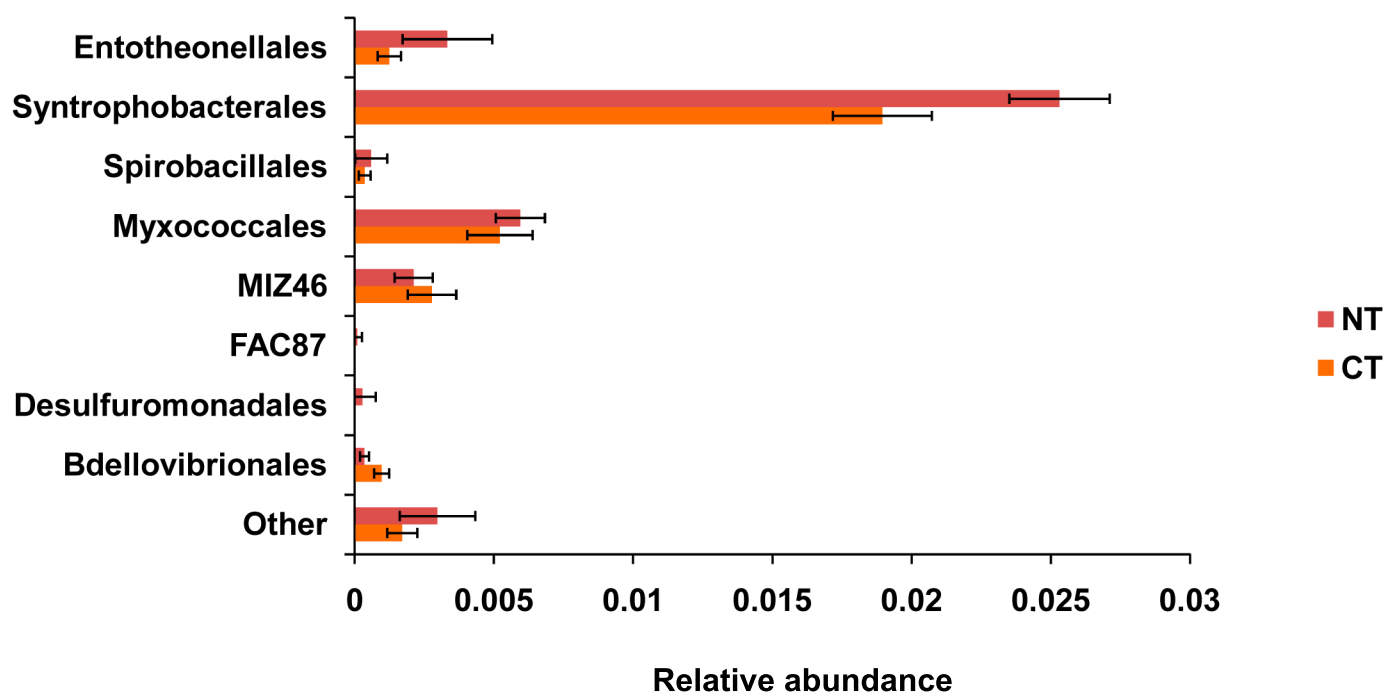

Supplement: Figure S4 — Relative abundances of reads assigned to orders within classes Gammaproteobacteria and Deltaporteobacteria in NT and CT soils. (PDF) [file pone.0099949.s004.pdf]
